# Possible Formation of Mitochondrial-RNA Containing Chimeric or Trimeric RNA Implies a Post-Transcriptional and Post-Splicing Mechanism for RNA Fusion

**DOI:** 10.1371/journal.pone.0077016

**Published:** 2013-10-24

**Authors:** Wei Yang, Jian-min Wu, An-ding Bi, Yong-chang Ou-yang, Hai-hong Shen, Gung-wei Chirn, Jian-hua Zhou, Emily Weiss, Emily Pauline Holman, D. Joshua Liao

**Affiliations:** 1 Guangxi Veterinary Research Institute, Nanning, Guangxi, P.R. China; 2 Hormel Institute, University of Minnesota, Austin, Minnesota, United States of America; 3 School of life Sciences, Gwangju Institute of Science and Technology, Gwangju, Korea; 4 Biomedical Engineering Department, Boston University, Boston, Massachusetts, United States of America; 5 Nantong University, Nantong, JianSu, P.R. China; Wayne State University, United States of America

## Abstract

Human cells are known to express many chimeric RNAs, i.e. RNAs containing two genes' sequences. Wondering whether there also is trimeric RNA, i.e. an RNA containing three genes' sequences, we wrote simple computer code to screen human expression sequence tags (ESTs) deposited in different public databases, and obtained hundreds of putative trimeric ESTs. We then used NCBI Blast and UCSC Blat browsers to further analyze their sequences, and identified 61 trimeric and two tetrameric ESTs (one EST containing four different sequences). We also identified 57 chimeric, trimeric or teterameric ESTs that contained both mitochondrial (mt) RNA and nuclear RNA (nRNA), i.e. were mtRNA-nRNA fusions. In some trimeric ESTs, the downstream partner was fused to the poly-A tail of the upstream partner, which, together with the mtRNA-nRNA fusions, suggests a possible new mechanism for RNA fusion that occurs after both transcription and splicing have been terminated, and possibly outside the nucleus, in contrast to the two current hypothetical mechanisms, trans-splicing and transcriptional-slippage, that occur in the nucleus. The mt-sequences in the mtRNA-nRNA fusions had pseudogenes in the nucleus but, surprisingly, localized mainly in chromosomes 1 and 5. In some mtRNA-nRNA fusions, as well as in some ESTs that were derived only from mtRNA, the mt-sequences might be cis- or trans-spliced. Actually, we cloned a new cis-spliced mtRNA, coined as 16SrRNA-s. Hence, mtDNA may not always be intron-less. Fusion of three or more RNAs to one, fusion of nRNA to mtRNA, and cis- or trans-splicing of mtRNA should all enlarge the cellular RNA repertoire, in turn enlarging the cellular functions. Therefore, future experimental verification of the existence of these novel classes of fusion RNAs and spliced mtRNAs in human cells should significantly advance our understanding of biology and medicine.

## Introduction

**Figure 1:**
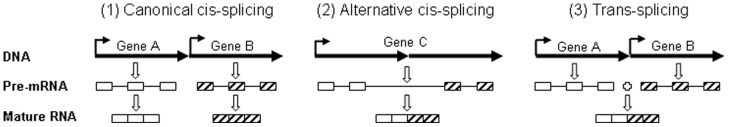
Our definition of cis- and trans-splicing in the situation of transcripts from two tandem genes on the same chromosome. (**1**): Two (A and B) tandem genes are transcribed separately as different pre-mRNA molecules, and each pre-mRNA is spliced independently to produce a mature RNA. This is canonical cis-splicing. (**2**): Transcription of gene A reads through the termination signal, due to whatever reason, and enters into gene B, resulting in a single, long pre-mRNA that can be regarded as: a) a transcript of a different gene (gene C), b) an alternatively terminated pre-mRNA of gene A, or c) an alternatively initiated transcript of gene B. This long pre-mRNA may be spliced to a different mRNA, such as one lacking the last exon of gene A and the first exon of gene B. We define this type of splicing of an alternatively transcribed pre-mRNA as an alternative cis-splicing. (**3**): Genes A and B are transcribed separately, and the two pre-mRNAs are spliced into one mature mRNA. We define this situation, if it happens, as trans-splicing. Hence, trans-splicing is a biochemical reaction involving two RNA transcripts as the substrate molecules, no matter whether they are transcribed from tandem genes on the same chromosome, from both Watson and Crick strands of the same genomic locus with a sense-and-antisense relationship, or from different chromosomes as two unrelated RNAs.

The swift spread of high throughput RNA sequencing technology in recent years has led to identification of tens of thousands of putative chimeric RNAs, which generally are considered as RNA molecules containing two genes' sequences. While a few hundreds of these chimeras are known to be transcribed from fusion genes that are formed due to genetic alterations, such as chromosomal rearrangements and genomic DNA deletion or amplification [1], for the vast remaining majority, how they are formed is still unknown. Trans-splicing may be one mechanism, but in mammalian cells it has not yet been well defined, in part because in mammals it is considered a rare event with a very different mechanism from that in low-level organisms [2]. Considering that the well characterized cis-splicing is a biochemical reaction that involves only one pre-mRNA as the substrate molecule and produces one mature mRNA, we define trans-splicing as a biochemical reaction that involves two RNA molecules as the substrates, no matter whether the product RNA codes for a protein or not. In other words, “one or two substrate RNA molecules” is a clear demarcation between cis- and trans-splicing, as illustrated in figure 1. The two substrate RNAs can be two copies of the same one; in this case trans-splicing often results in an RNA with duplicated exons, such as the 77–80 kD variant of human estrogen receptor alpha [3], [4]. The two substrate RNAs can also be a sense and an antisense transcribed from the same genomic locus, and can be pre-mRNA of two different genes on the same chromosome or on different chromosomes. In a report from the ENCODE, it is estimated that RNA transcripts from about 65% of the human genes form chimeric RNA with a transcript from another gene, but in most cases this other gene is nearby on the same chromosome [5], [6]. However, some of those chimeras formed between two tandem genes on the same chromosome may be derived from a single RNA transcript and thus are not real chimeras, by our definition. Instead, such a long transcript spanning two tandem genes can be considered as 1) an RNA product of a different, not-yet-annotated, gene, 2) an alternatively terminated transcript of the first gene, or 3) an alternatively initiated transcript of the second gene. This long transcript may undergo a different cis-splicing, producing a mature RNA product, as illustrated in figure 1. Unfortunately, of those chimeras reported by the ENCODE or deposited in different public databases, it is unclear which ones are processed from a single RNA precursor and which other ones result from two separate precursors.

A significant percentage of putative chimeric RNAs encompass a short homologous sequence (SHS) shared by the two partners [7]. Although its reason is unknown, this feature has led to a so-called “transcriptional-slippage” hypothesis on how this type of chimeras are formed, which infers that two genes are in the same transcriptosome and transcription slips from one gene to the other, due to the complementarity at the SHS [7]. Since transcription of chromosomal DNA occurs in the nucleus and splicing occurs while the transcription is still elongating [8], both the transcriptional-slippage and the trans-splicing, if they indeed happen, should occur in the nucleus.

Since two RNA molecules could fuse to one, we wondered whether three RNAs could also fuse to one. In this study, we identified some expression sequence tags (ESTs) that contained three genes' sequences, thus coined as trimeric ESTs or trimeras. We also identified some ESTs that contained both mitochondrial (mt) RNA (mtRNA) and nuclear RNA (nRNA). In some ESTs, the downstream partner was fused to the poly-A tail of the upstream partner. Since mtRNA are not transcribed in the nucleus and polyadenylation occurs after both transcription and splicing are terminated, these findings are inklings of the existence of a previously unsuspected mechanism for RNA fusion, besides the hypothetic trans-splicing and transcriptional-slippage. The possible existence of trimeras and mtRNA-nRNA fusions in human cells suggests that the whole cellular RNA repertoire may be much larger than what we have known, which in turn enlarges cellular functions.

## Results

### There are trimeric and tetrameric ESTs

**Figure 2:**
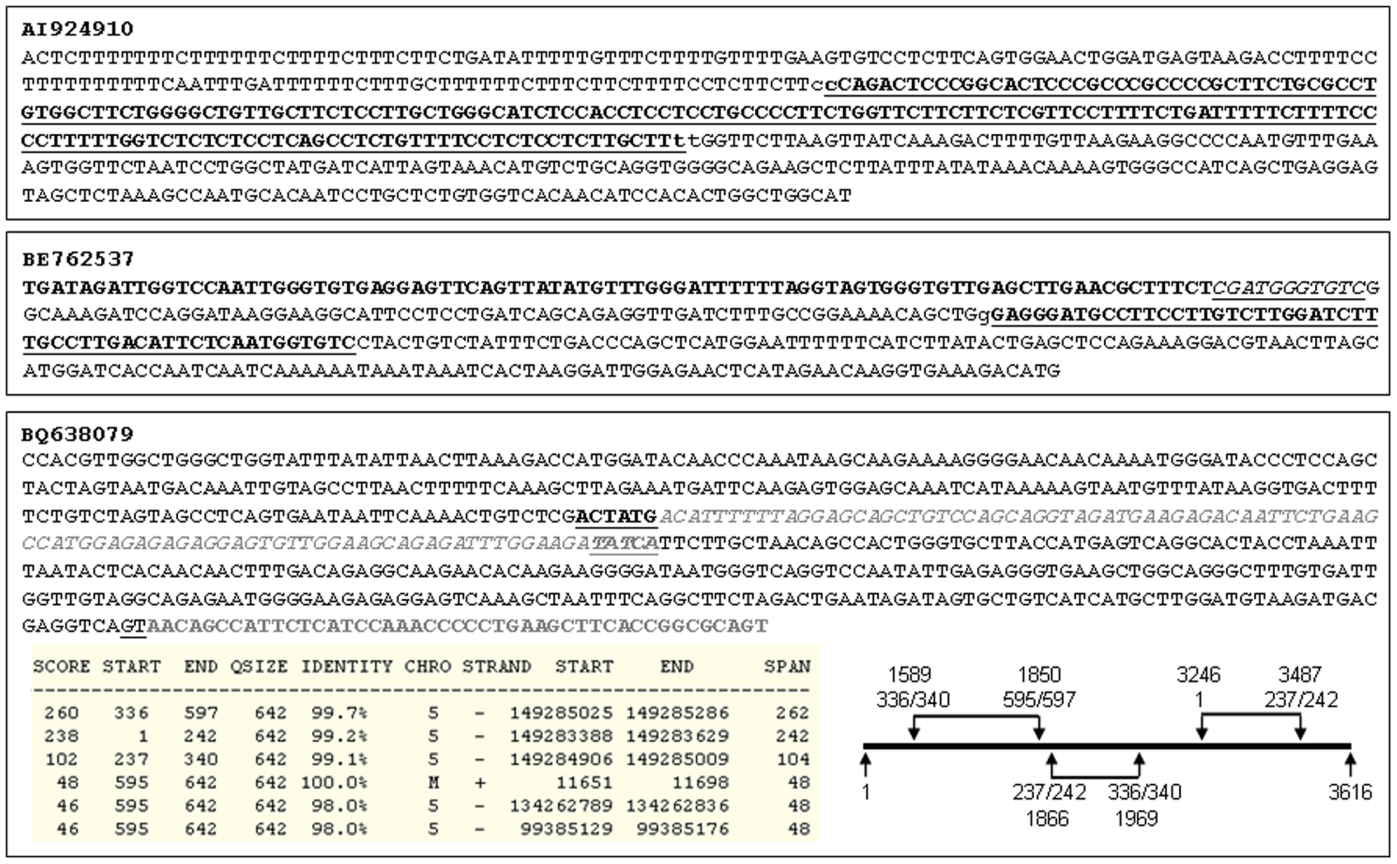
Examples of trimeric and tetrameric ESTs with or without an mt-sequence. **Top panel**: AI924910 is a trimera. Its 1–156th, 155–342th, and 341–550th nt regions match, respectively, the 5468–5676th nt region of the CYCO1 mRNA from chromosome 3, the 4332–4518th nt region of the PDCD11 mRNA from chromosome 10 (underlined and boldfaced), and the 366–521st nt region of the SREK1IP1 mRNA from chromosome 5. Between the two neighboring partners, there are two nt (lowercase letters) overlapped. **Middle panel**: BE762537 is a tetramera. Its first 86 nt (boldfaced) match the 2198–2283rd nt of the L-strand of mtDNA. The following 11-nt sequence (italicized and underlined) is an unmatchable gap. Its 98–168th nt sequence is part of the alternatively spliced exon 2 of the UBC mRNA from chromosome 12, which is followed by a 53-nt (the 268–220th) UBC antisense fragment (boldfaced and underlined). Both UBC sense and antisense fragments, which overlap at the 168th nt (the lowercase “g”), have multiple repeats in the UBC mRNA (not shown). The last 149-nt (the 221–369th nt) sequence is part of the ENPP6 mRNA from chromosome 4. **Bottom panel**: BQ638079 is a 642-bp tetrameric EST. Its first 595–597 nt sequence consists of three fragments from intron 7 of the PDE6A gene on the minus strand of chromosome 5. The relationships among these three fragments within this 3616-bp intron are illustrated in the figure, with the 3-digit numbers indicating the 5′ or 3′ end of each fragment and the 4-digit numbers indicating the position at the intron. As shown in the sequence, the figure, and a table copied from the UCSC browser, the middle fragment is 104-bp long, is matched to the 1866–1969th nt region of intron 7, and shares 6 or 5 nt (the underlined and boldfaced 237–242nd or 336–340th nt) with its up- or down-stream fragment that actually comes from the 3′ or the 5′ part, respectively, of the intron 7. The last 46–48 nt sequence of this EST come from the H-strand of mtDNA, with 2 nt (underlined GT in the sequence) overlapped with the last fragment of intron 7 of the PDE6A. The mt-sequence has two highly homologous NUMTs on chromosome 5, as shown in the table.

We designed simple computer code to screen preliminarily EST and mRNA collections in the NCBI database and obtained hundreds of putative trimeric ESTs. We then used the NCBI Blast and UCSC Blat browsers to analyze the sequences of these ESTs and identified 61 trimeric ESTs (table 1), such as AI924910 (Fig. 2). We also found that two ESTs, i.e. BE762537 and BQ638079 (Fig. 2), contained four sequence components, coined as tetrameric RNAs or tetrameras. There are many more ESTs that have passed our initial screening but have not yet been analyzed using Blast and Blat; hence, many more trimeric and tetrameric ESTs are anticipated.

**Table 1:** Trimeric ESTs.

AI335862	BF826714	BF826602	BF764896	BF762577	BF744644	BF331329	BF306729	BE694080
AI924910	AU132130	BF109407	AV744183	AV729389	AV725012	BE814336	BE762537	BE876577
AW608255	BE715872	BE715869	BE715858	BE709675	BE694009	BE696199	BQ348968	AA514694
AW956968	BF803049	BF764896	BF331329	AU142287	BE172179	X93499	BM824189	BG995785
AW999004	BQ689257	BQ689139	BU539467	AI925024	BF814512	BG003110	R19361	BE898652
BC064904	M77198	BM915020	BI004882	BF995070	BF878278	BF109407	AW994480	BE716966
BE074730	BE876742	BE937759	BF987118	BM691077	BM703781	BX109950		

Note: Each of these ESTs contains three sequence elements.

**Figure 3:**
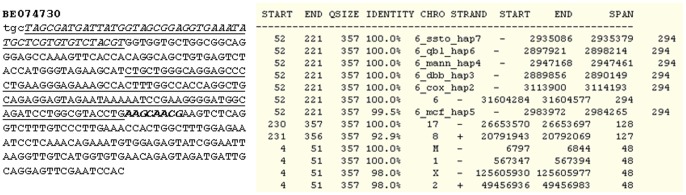
BE074730 is a trimera. The origin of its 4–51st nt sequence (italicized and underlined) is unclear because it is completely matched not only to mtDNA but also to an NUMT on chromosome 1, as shown in the table copied from the UCSC browser. Its 52–221st nt sequence consists of two exons of the PRRC2A (also called BAT2) mRNA, with the downstream exon underlined; its real genomic origin is unclear, because this nRNA sequence belongs to a cluster of genes on chromosome 6 as shown in the table. The 230–357th nt sequence of this EST is part of the TMEM97 mRNA from chromosome 17. Actually, the 4–51st sequence also has homolog on chromosomes 2 and X, while the 230–357th sequence has homolog on chromosome 8. There is an 8-nt (boldfaced and italicized AAGCAACG) unmatchable gap between the 2nd and the 3rd partners.

Some trimeras or tetrameras, such as BQ638079, contained two or three nRNA elements that were derived from the same chromosomal locus but were not linear, i.e. not a cis-spliced product (Fig. 2); likely, trans-splicing was involved in formation of these fusion RNAs, if they were not artifacts. In many of these multi-component ESTs, at least one sequence element had multiple locations in the genome, either on the same chromosome or on different chromosomes (table 2), making it impossible to determine its true origin. For example, the first component, i.e. the most 5′ partner, of the AV725012 had tens of locations on quite a few chromosomes (data not shown), whereas in the BE074730 (Fig. 3) and BF109407, an nRNA element had several copies on the same chromosome. Some of the trimeras have putative open reading frame for protein translation, as analyzed using DNAstar software, but the open reading frame cannot be fully analyzed because most, if not all, ESTs have not yet been cloned for their full-length sequence.

**Table 2:** ESTs that contain a sequence with mulitple repeats in nuclear DNA.

AK098472	BF826602	BF826714	BF803049	BF764896	BF762577	BF744644	EG328081	BF331329
BE172179	BF306729	AU132130	BF109407	AV751897	BF083272	BF083248	AV744183	AV727918
BE149219	AV725012	AV704911	AV703546	AV702773	BE899559	BE899119	BE898652	BE876577
DB370064	AV696226	AV695866	AV691857	BE814336	BE762537	BE716966	BE715872	BE709354
DB324119	BE709292	BE696199	BE694009	AV696226	BF803049	BF764896	BF378297	BE898652
DB277685	BF331329	BF185864	AU142287	BE898652				

Note: At least one of the sequense element in these ESTs has multiple copies in nuclear DNA.

### nRNA may be fused to mtRNA

We also identified 57 ESTs that were nRNA-mtRNA fusion, i.e. contained both nRNA and mt-sequence (table 3). In most of these ESTs, the mt-sequence had one or more pesudogenes in the nucleus, which were more often referred to as NUMT (nuclear mitochondrial sequence) [9], [10]. These 57 ESTs together contained a total of 74 NUMTs, of which 32 (43.2%) and 25 (33.8%) were localized in chromosomes 1 and 5, respectively, with the remaining 23% in other chromosomes.

**Table 3:** Chimeric, trimeric or tetrameric ESTs that contain mitochondrial sequence.

	Mitochondria			Chromosome		Fusion
#	Access #	M strand/region	M-span (nt)	NUMT	Partner	
1	DB324119.1	L7212–7515	304	1	M-10	Chimera
2	BE898652.1	L8462–8527, L8569–8969	62, 401	1, 1	M-M-19	Trimera
3	AU142287.1	H4547–4895	349	1	22-M-5	Trimera
4	BF378297.1	L8443–8642	200	1	M-22	Chimera
5	BE716966.1	H7587–7790	204	1	M-1–11	Trimera
6	BE899119.1	H8399–8936	538	1	M-22	Chimera
7	AV744183.1	H4333–4399	67	1	M-19–9	Trimera
8	BF762577.1	L6897–7014, L9645–9779	118,135	1, 1	7-M-M	Trimera
9	BF764896.1	H11165–11248	84	5	2-M-6	Trimera
10	BE709292.1	L10705–10865	161	5	M-7	Chimera
11	BE709354.1	L10705–10864	160	5	M-7	Chimera
12	BE899559.1	H10677–1170	494	5	6-M	Chimera
13	BF083248.1	H14061–14231	171	5	10-M	Chimera
14	BF083272.1	H14061–14233	173	5	10-M	Chimera
15	AV751897.1	H10511–10874	364	5	M-19	Chimera
16	BF306729.1	H10637–10864	227	5	5-M	Chimera
17	BF744644.1	H15540–15721, H14092–14295	182, 204	5	M-M-11	Trimera
18	BE762537.1	L2198–2283	86	17	M-12–12–4	Tetramera
19	BE876577.1	H10689–11361, H84455–8680	493, 226	5, 1	M-M-11	Trimera
20	AV702773.1	H2639–3082	444	17	14-M	Chimera
21	AA514694	L9176–9208	33	1	M-8–8	Trimera
22	AA581515	L7399–7453	55	1, 17	M-17	Chimera
23	AA679609	L7399–7520	122	1	M-22	Chimera
24	AA679609	L7399–7520 (≈AA679609)	122	1	M-22	Chimera
25	AI925024	H2037–2239	203	3, 11, 5	14-M-13	Trimera
26	AW134795	L1619–1671	53	11	M-1	Chimera
27	AW370799	H8966–9070	105	1, 5	19-M	Chimera
28	AW753072	L14400–14455	56	18, 5, 17, 5	M-9	Chimera
29	AW821349	L12015–12081	67	5	M-16	Chimera
30	AW898803	H2647–2773	127	None	X-M	Chimera
31	AW950200	H7276–7520	245	1	5-M	Chimera
32	BE074730	L6707–6844	48	1, x, 2	M-6–17	Trimera
33	BE162186	H5117–5322	205	1	M-13	Chimera
34	BE876742	H7398–7457	60	1	19-M-1	Trimera
35	BE937759	L9080–9144	65	1	9-M	Chimera
36	BF852160	L4703–4822	120	1	17-M	Chimera
37	BF987118	H6568–6604	37	1	M-21–7	Trimera
38	BF988359	L6876–6938, L6950–7025	63, 76	1, 1	7-M-M	Chimera
39	BG995785	H10473–10589, L15168–15217	117, 50	5, 5	M-M-1	Trimera
40	BM691077	H14103–14159	57	5	17-M-1	Trimera
41	BM703781	H9567–9605	39	1	4-M	Chimera
42	BM997144	L7189–7520	332	1	M-2	Chimera
43	BP348380	H2852–3018	167	5	19-M	Chimera
44	BQ300150	H8936–8995	60	1	3-M	Chimera
45	BQ348968	L12338–12505	168	None	M-8–2	Trimera
46	BQ638079	H11651–11698	48	5, 5	5–5–5-M	Tetramera
47	CV385666	L2620–2837	218	11	M-7	Chimera
48	DA086571	H7201–7521	321	1	M-1	Chimera
49	DA182598	H2417–2733	317	11,3,6,17,5	M-11	Chimera
50	DA365070	H1613–1672	60	11, 7, 5	M-7	Chimera
51	DA511096	H7192–7533	342	1	M-1	Chimera
52	DA757571	H2421–2762	342	None	M-1	Chimera
53	DB314922	L7393–7515	123	1	M-1	Chimera
54	DB324119	L7212–7515	304	1	M-10	Chimera
55	AW994480	L2654–2804, L2814–2880, L2894–2984	63, 67, 91	None	M-2–8	Trimera
56	BE694080	L2654–2984 (≈AW994480)	63, 67, 91	None	M-2–8	Trimera
57	BF826602	L11053–11116	64	5, 5	12–15-M	Trimera

Note: The first and last nt positions at the mtDNA of an mt sequence (M) are indicated, based on UCSC browser, while its length (span in the number of nt) may not always be calculated due to possible deletion of several nt. The chromosome or chromosomes that harbor an NUMT homologous to the mt sequence are indicated. The order of each partner in the chimera, trimera or tetramera is shown in the 5′-to-3′ orientation.

**Figure 4:**
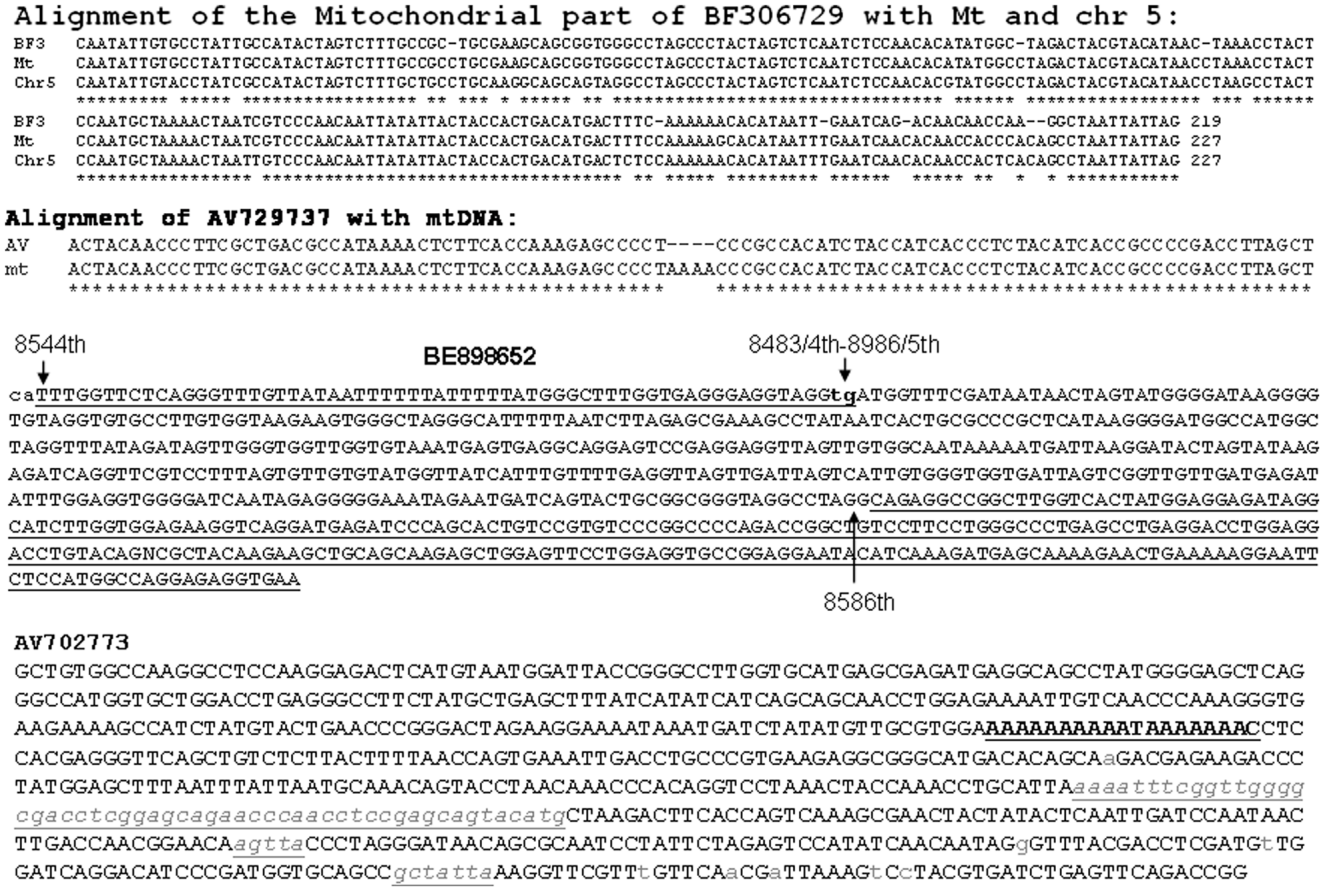
Cis- or trans-spiced mtRNA in ESTs. Alignment of the mt-part of EST BF306729 (BF3) with mtDNA (mt) and a corresponding NUMT on chromosome 5 (Chr 5) shows that the EST matches better to the mtDNA than to the NUMT, suggesting that the mt-sequence is more likely to be transcribed from mtDNA, but not its nuclear counterpart. Mismatched positions are often deletions. AV729737 is an mt-only EST that has an “AAAA” deletion when aligned with mtDNA. BE898652 is a trimera containing trans-spliced mt-sequence. Its 3–64th nt region is matched to the L-strand of an mtDNA (AC_000021), which is part of the ATP8 gene's antisense, while its 63–461st nt sequence is matched to the 8586–8986th nt of the L-strand, which is part of the ATP6 gene's antisense. The two sequences overlap at the boldfaced lowercase “tg”. The last part of this EST (underlined) is part of the PSMC4 mRNA from chromosome 19. AV702773 is a chimera. Its 1–245th nt region belongs to the last 3 exons of the PSME2 mRNA from chromosome 14, which is followed first by a poly-A signal (the 246–263rd nt, containing a T) and then by an mt-sequence (265–708th nt) from the 16S rRNA gene (the 2639–3082nd nt of mtDNA). However, since the PSME2 mRNA (NM_002818.2) in the NCBI database does not have a poly-A signal or a poly-A tail, it is unclear whether this poly-A is part of PSME2 that is undocumented or is part of the following mtRNA that is polyadenylated. Note that one long and two short italicized lowercase sequences in grey within the mtDNA region do not actually exist in, i.e. are deleted from, AV702773. Several single lowercase letters in grey are mismatches to the mtDNA (AY195786.2).

Most of these mt-sequences were better matched to the mtDNA than to their nuclear counterparts, as exemplified by BF306729 (Fig. 4), and thus were more likely from the mitochondria than from the nucleus. At the positions where the mt-sequence was mismatched to the mtDNA or the corresponding NUMT, the mismatch was more often a deletion, as shown in BF306729 (Fig. 4). mtRNAs are known to be subjected to RNA editing, non-template 3′ addition, base modifications and other different posttranscriptional modifications [11], [12], making its cDNA sequence often mismatched to the mtDNA and NUMT. Therefore, sometimes it is difficult to determine the origin of an mt-sequence in the ESTs by sequence alignment. On the other hand, there also were some ESTs in which the mt-sequence was identical to the corresponding NUMT, such as in the BE074730 (Fig 3); in this situation it is unsure that the mt-sequence is derived from the mtDNA, although currently there is little evidence suggesting that NUMTs are stably expressed. Several ESTs had a reoccurrence with identical sequence, which enhances their fidelity.

### mtRNAs may undergo cis- and trans-splicing

Some chimeric and trimeric ESTs listed in table 3 that contained an mt-sequence showed one or several deleted regions that were set arbitrarily as 4 nucleotides (nt) or longer, such as in the AV702773 (Fig. 4), when aligned with mtDNA sequence. We also identified 12 ESTs, such as the AV729737 (Fig. 4), that contained only mt-sequence but the sequence had such deletions. Two of these 12 ESTs had reoccurrence (table 4). There were many other ESTs that were not counted because the deleted part was 3 nt or less.

**Table 4:** ESTs that are mt-only sequences and are probably cis-spliced products.

EST	linear mt regions	EST	Linear mt regions
BE701368	H887–948, H1049–1108	BE932324	L651–1152, L1162–1218, L1223–1246
BE162186	H5117–5153, H5158–5322	BE932352	Reoccurence of BE932324
BG429734	multiple 4–14 nt gaps	BE875084	H5616–5792, H5800–5966, H5915–6171
BP348380	H2852–2921, H2829–3018	BE876577	H10869–11100, H11107–11361
AA070765	H1755–1881, H1888–2157	AV729737	H3017–3489, H3494–3721
AW994480	L2654–2804, L2814–2880, L2894–2984	BF676688	H4333–4912, H4933–5037
BE694080	Reoccurence of AW994480	BF988359	L6876–6938, L6950–7025

Note: These ESTs consist of only mt sequences but with one or more deletions of 4-nt or more, thus being cis-spliced products.

In four mt-only ESTs, the mt-sequences were not linear along the mtDNA, thus likely being trans-spliced products. Besides the light (L) strand containing BE898652 shown in figure 4, the other three were the heavy (H) strand containing BF744644 and BF876577 and the BG995785 that contained both H- and L-strand sequences.

### Some fusion ESTs contain poly-A between the two partners

**Figure 5:**
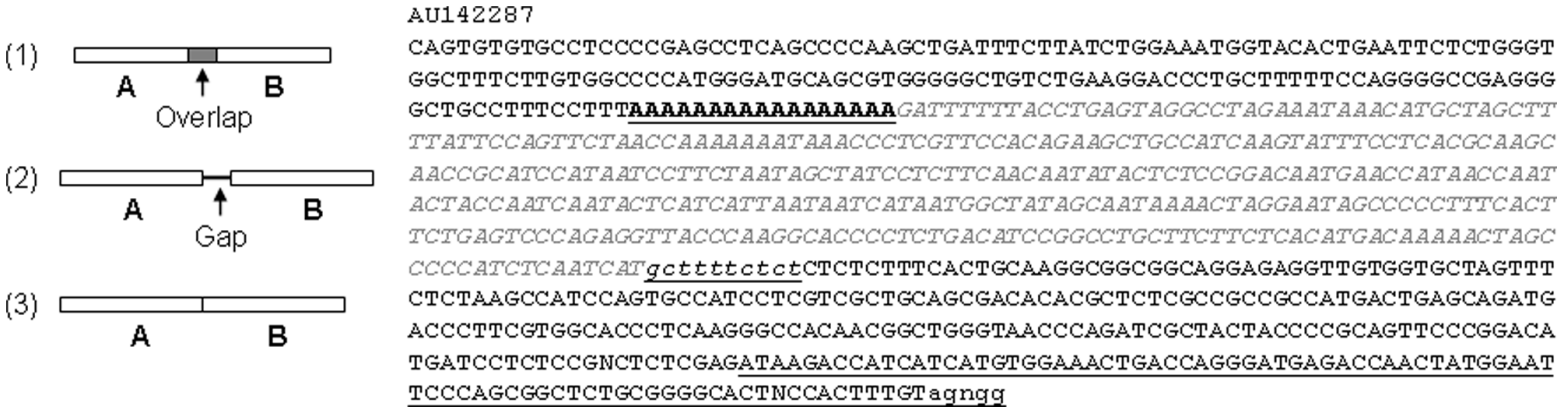
Poly-A as the gap between two fusion partners. **Left panel**: In a fusion RNA, two neighboring genes' sequences have three relationships, i.e. 1) with an overlapped sequence, 2) with an unmatchable sequence as a gap, or 3) directly joined. **Right panel**: The 1–160th nt of EST AU142287 are matched the 429–588th nt of the last exon of the POLDIP3 mRNA from chromosome 22, followed by a poly-A tail (boldfaced and underlined). Since the wild type form of this exon should have 2248 nt, the polyadenylation actually follows an early transcriptional termination. The 178–526th nt region (italicized grey) after the poly-A is part of the ND2 mRNA from mtDNA. The 527–836th nt sequence belongs to the first two exons of the GNB2L1 mRNA from chromosome 5, with the first 10 nt (underlined lowercase letters) alternatively initiated from the −10 bp of the GNB2L1 gene. The last 85 nt (underlined) have a few deletion mismatches to the first 88 nt of exon 2 of the GNB2L1. The last 5 nt (agngg) are unmatchable and might belong to the cloning vector.

In a fusion RNA, the two neighboring partners have three different relationships (Fig 5A), i.e. 1) with a sequence overlapped by the two partners, 2) with an unmatchable sequence as a gap, or 3) directly joined without a gap or an overlap. The frequency with an overlapped sequence was much higher than that with a gap, while the frequency for joining directly was the lowest (Table 5). In most ESTs in the databases we analyzed, the overlapped sequence was short, with 3–5 bp as the medium (table 5), just as coined by Li et al previously as SHS [7]. The gap sequence was also short in most cases, although much longer than the overlapped sequence (table 5). For unknown reasons, the gap was still unmatchable to any genomic region of human or any other organisms even when its length was long enough to be specific. Because it is unmatchable, it is regarded as a gap sequence, but not as an additional fusion element. Actually, we sometimes obtained such unmatchable sequence data during our cloning human cDNAs. If the hypothetical 3′-to-5′ inverse but not complementary sequences exist [13], [14], it may be a possible explanation.

**Table 5:** Frequencies of three different relationships between two neighboring parnters in chimeric, trimeric and tetrameric RNAs.

Database	Relationship	N	Mean	Median(Q2)	Maxima(Q4)	Q1	Q3	Sstdv	Mad
mRNA in NCBI	Exact junction	157							
	Overlapped	736	6.5	3	606	2	6	29.52	2.97
	Gap (insert)	373	132.4	49	1303	13	167.5	198.79	65.23
EST in NCBI	Exact junction	2520							
	Overlapped	23252	6.4	5	190	3	7	7.7	2.97
	Gap (insert)	8998	29.5	21	307	8	38	31.4	20.76

Note: Putative chimeric, trimeric and tetrameric RNAs were identified from mRNA and EST collections of the NCBI database by our simple computer code. “Exact junction” means that the two partner mRNAs join directly. “Overlapped” means that the two partner mRNAs have at least 1 nucleotide (nt) overlapped. “Gap (insert)” means that there is at least 1-nt or an unmatchable sequence inserted between the two partner RNAs. The length of the overlapped or inserted sequences is short in most cases and is not normally distributed. Therefore, the mean, midian (Q2) and maximal lengthes in number of nt are calculated, besides the length at the 25%th (Q1) and 75% (Q3). Mad(X)  = 1.4826 * median (|Xi-median(X)|). * When the distribution is so different from normal distribution, we usually compare “mean” to “median” and “standard deviation” to “mad”. If the difference between mean and median, STDEV and MAD are huge, the distribution is far from normal distribution.

Intriguingly, in some ESTs the gap was actually a poly-A sequence, or a poly-T when the upstream partner was reverse-complementary to the mRNA, indicating that the downstream partner was fused to the poly-A tail of the upstream partner, such as in the AU142287 in which the mt-sequence was fused to the poly-A tail of the upstream nRNA (Fig 5). The poly-A or poly-T was usually appended to an earlier termination, but not the canonical end, of the last exon, such as in the AW608255 and AU142287. In some other cases such as the BM691077, the poly-A was appended at the 3′ end of the mtRNA sequence; more likely, the polyadenylation of the mtRNA occurred before fusion by the nRNA element.

### Identification of 16SrRNA-s, a cis-spliced mtRNA

**Figure 6:**
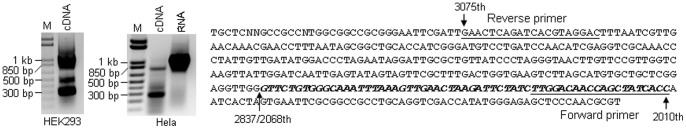
Expression of 16SrRNA-s. RT-PCR with primers that amplify the 2010-3075th region of mtDNA detects a 300-bp band and a 500-bp band, besides the anticipated band at about 1-kb, in HEK293 cells. The 500-bp band occurs randomly with greatly variable density, thus likely being a heterodimer of the 300-bp and 1-kb cDNAs, which appears randomly and is a common phenomenon as we have frequently described [37], [39], [40]. Isolation of the 300-bp band followed by T-A cloning and sequencing reveals that it is an mtRNA with a 768-bp (the 2069–2836th nt region of mtDNA) deletion, as indicated in the sequence. The boldfaced sequence is the downstream exon. The number of the nt is based on the UCSC browser, with the position of the last nt in the reverse primer (underlined) and the first nt in the forward primer (underlined) indicated. The sequences outside the primers belong to the T-A vector. Because the RNA sample was not pre-digested with DNase, the 1-kb band should be derived from not only cDNA, but also mtDNA, of the 16S rRNA gene. Removal of DNA from an RNA sample from Hela cells followed by RT-PCR (cDNA) still detects the 300-bp band, besides a band at about 850-bp, while the 1-kb band was very weak. Cloning and sequencing the 850-bp band reveal that it is part of the 16S rRNA that was amplified because the reverse primer is also partly reverse-complementary to the mtDNA. Direct PCR amplification of the RNA sample without RT (RNA) detects only the 1-kb band, but not the 300-bp one, indicating that the 300-bp band is not associated with an mtDNA with deletion.

We performed reverse transcription (RT) and polymerase chain reaction (PCR) followed by T-A cloning and sequencing to verify those trimeric or terameric ESTs shown in figures 2, 3, 3, 5, i.e. the AI924910, BE762537, BQ638079, BE074730, BE898652, AV702773 and AU142287, using total RNA sample from the whole-cell lysate of HEK293 cells, but we failed to detect any of these seven ESTs. However, we serendipitously identified a cis-spliced mtRNA in HEK293 cells, which had a 769-bp, i.e. the 2067–2836^th^ nt region of the mtDNA, deleted from the 16S rRNA, thus coined as 16SrRNA-s (Fig 6). The same mtRNA was also expressed in Hela cells, as confirmed by RT-PCR followed by T-A cloning and sequencing. PCR of total RNA sample from Hela cells without RT amplified the mtDNA, but not this 16SrRNA-s, indicating that it is not associated with an mtDNA with deletion (Fig. 6). Because it was detected by RT-PCR that lacks the strand-specificity, as described by us recently [15], it is unclear whether this novel mtRNA is transcribed from the H- or the L-strand of the mtDNA.

## Discusion

### Human cells may have trimeric or tetrameric RNAs

This study reports, for the first time, the existence of trimeric and tetrameric ESTs. Although our RT-PCR studies failed to confirm the expression of seven of these fusion RNAs in HEK293 cells, it cannot be excluded that these seven and other trimeras and tetramers are expressed in other situation or in other cell types. How the three or four different sequences are fused into one is unknown. It remains possible that the mechanism for formation of chimeras, trimeras and tetrameras is similar, no matter whether the fusion occurs as a cellular event or as a technical artifact.

### Is mtRNA-nRNA fusion a mechanism to enlarge the RNA repertoire?

This study is also the first report on ESTs that are mtRNA-nRNA fusions. If this type of fusion truly exists and if the involved mt-element is truly derived from mitochondria, but not from its nuclear counterpart (i.e. NUMT), it suggests that two different pools of RNA, i.e. nRNA and mtRNA, can join together to constitute a previously unaware mechanism to enlarge the cellular RNA repertoire. Since most part of the transcript from the L-strand is known to be non-coding, it is possible that some parts of the L-strand transcript may be used as a resource for mtRNA-nRNA fusion to enlarge the ensemble of the cellular RNA, which is supported by a recent report of the existence of mtRNA antisenses [16]. Similarly, we found that the nRNA components in many ESTs were large and did not belong to exons of known genes. Likely, contribution to mtRNA-nRNA fusions may be a previously unsuspected role of non-coding nRNAs as well.

Considering that mtDNA encompasses only about 16.5 kb whereas the human genome contains 3164.7 million base-pairs (bp) (www.ornl.gov/sci/techresources/Human_Genome/project/info.shtml), it seems that mtRNA has a much higher frequency than nRNA to form fusion RNA, no matter whether the fusions are genuine or spurious. This may partly be because a set of processed mtRNAs are not polyadenylated [17], leaving them a higher chance to form spurious chimeras as we inferred recently [15].

### mtRNA-nRNA suggests a new mechanism for RNA fusion

Tens of thousands of chimeric RNA have been identified so far, but the vast majority of them still remain putative, waiting for verification of their true existence and determination of how they are formed. For those true chimeras that are formed at the RNA level, i.e. are not transcribed from a fusion gene, transcriptional-slippage and trans-splicing are the two major hypothetical mechanisms. However, if there are mtRNA-nRNA fusions, even if there is just a single one, it implies that either nRNAs are transported into the cytoplasm, likely into the mitochondria, to fuse with mtRNA, or mtRNAs are transported into the nucleus to fuse with nRNAs. Appearance of mtRNA in the nucleus has been reported [18], but it is technically difficult to confirm because the nuclear genome contains as many as 755 NUMTs [9], [10]. Appearance of nRNA in the mitochondria has also been reported [12], [16], [19], [20], although more studies are needed to confirm that large nRNAs are as easy as microRNA to relocate to the mitochondria. No matter which one goes into which, it indicates a previously unknown mechanism for RNA fusion, besides the transcriptional-slippage and trans-splicing, because transportation of nRNA to the mitochondria or fusion of the mtRNA to the nRNA should occur after the nuclear transcription has been terminated and splicing has been completed. The identification of those ESTs in which the downstream partner is fused to the poly-A tail of the upstream partner also supports a “post-transcription and post-splicing” mechanism, because polyadenylation occurs after both RNA transcript cleavage and splicing completion, although the polyadenylation may continue in the cytoplasm [21]. Moreover, this new mechanism may occur outside the nucleus, whereas both transcriptional-slippage and trans-splicing of nRNA can only occur in the nucleus.

### Do mtRNAs also undergo cis- and trans-splicing?

RNA editing as well as cis- and trans-splicing are known to occur in mtRNAs of plants and some low-level organisms [22]–[25]. However, to our knowledge there has only been one reported chimeric mtRNA in the human [26], [27] and another one in the mouse [28]. Human mtDNA does not contain introns and thus human mtRNAs are not supposed to undergo cis-splicing [17]. Therefore, our identification of the ESTs that contain two or more linear but segregated mt-sequences is a surprise, as it suggests that human mtRNA may also undergo cis-splicing. Such events may occur more frequently in disease situations since most ESTs are cloned from cancer cell lines, and since over 400 mutations or mtDNA rearrangements or deletions have been identified in different human diseases [29]–[31]. In the human mtRNA EU863789 identified by Burzio et al [26], the 2810–3125^th^ nt region of the H-strand is trans-spliced to the 1673–3227^th^ nt region of the L-strand, creating a 1866-nt chimeric RNA with its 311–316^th^ nt region as a SHS shared by the two elements, according to our analysis. The EST BG995785 also contains both H- and L-strand sequences and thus may be a trans-spliced product as well. Another trans-spliced EST we identified is the EB898652, which is formed by two L-strand sequences (Fig. 4). Although most ESTs may be resulted from RT-PCR related techniques and thus are poor in the strand-specificity as we described recently [15], the interrelationship of the two mt-sequences within an EST should not be changed. The most convincing evidence for cis-splicing of mtRNA is our identification of the 16SrRNA-s in both HEK293 and Hela cells, as it is not associated with a deletion in the mtDNA (Fig. 6).

### The mtRNAs that fuse with nRNA prefer to save copies in chromosomes 1 and 5

It is a surprising finding that the mt-sequences in mtRNA-nRNA fusions have their corresponding NUMTs mainly in chromosomes 1 and 5 (Table 3), because these two chromosomes do not have significantly more NUMTs than other chromosomes [9], [32]. Since evolutional insertion of mtDNA into the nuclear genome is not a random event [10], a common mechanism, which has a preference to chromosomes 1 and 5, may regulate both the fusion between some mtRNAs and some nRNAs and the insertion of the same mtRNAs into the nuclear genome. This hypothetical thinking deserves further exploration.

### Abundant mtDNA is an unvanquished obstacle for mtRNA detection

Because it is unclear whether the mtRNA-nRNA fusion occurs in the nucleus, cytoplasm or mitochondria, we determined the expression of seven fusion ESTs in the whole cell lysates with RT-PCR, but failed to confirm the existence of any of the seven in HEK293 cells. Study of mtRNA is technically more difficult than that of nRNA, partly because mtDNA is much smaller than chromosomal DNA and thus easier to mingle with cellular RNA. Moreover, normally one cell only has two sets of genomic DNA, although cancer cells are often of aneuploidy. In sharp contrast, one cell has from hundreds to thousands of mitochondria, and each mitochondrion contains multiple copies of mtDNA [33]–[35]. More complexly, many, but usually not all, copies of mtDNA may have mutations, deletions and other types of changes in cancer cells [29]–[31], which will likely cause additional bands in PCR amplification of mtDNA or mt-cDNA. Clearance of such huge amount of mtDNA by DNase followed by inactivation of the enzyme without causing RNA degradation is difficult, if not impossible. If DNase digestion is skipped, the mtDNA in the RNA sample will, to a large extent, compete out the cDNA of interest by depriving it from primers during PCR amplification of the RT products, which actually is somewhat reflected in figure 6. Moreover, because most mtRNA species are not supposed to undergo splicing and are thus identical in the length with their parental mt-genes, removal of mtDNA from the RNA sample is necessary in many situations, as we have discussed in more detail recently [36]. These technical constraints affect the RT-PCR detection of those mtRNA and mtRNA-nRNA fusions that are expressed at low abundance. We are trying to solve this technical hurdle so that we can better verify the expression of trimeric ESTs.

## Conclusions

Sequence analyses of ESTs deposited in the NCBI suggest the possible existence of trimeric and tetrameric RNAs and the existence of mtRNA-nRNA fusions. The involved mt-sequences had their NUMTs preferentially in chromosomes 1 and 5. In some chimeric or trimeric ESTs, the downstream partner is fused to the poly-A of the upstream partner, which, together with the mtRNA-nRNA fusions, suggests a post-transcriptional and post-splicing mechanism for RNA fusion that does not necessarily occur in the nucleus, unlike the hypothetical transcriptional-slippage and trans-splicing. Moreover, we also identified many ESTs in which the mt-sequence might be a cis- or trans-spliced product, and we cloned a novel cis-spliced mtRNA coined as 16SrRNA-s, which together suggests that mtDNA may not always be intron-less. Fusion of several RNAs into one, fusion of nRNA to mtRNA, as well as cis- or trans-splicing of mtRNA all should enlarge the whole cellular RNA repertoire, in turn diversifying the cellular functions.

## Materials and Methods

### Computational sequence analyses

We wrote simple computer code and used it to screen preliminarily the EST and mRNA collections in the NCBI database to identify putative trimeric RNAs. We then used NCBI Blast (http://blast.ncbi.nlm.nih.gov/Blast.cgi) and UCSC Blat browsers (http://genome.ucsc.edu/) to analyze the sequence of those putative trimeric ESTs. Since some of the chimeric, trimeric and tetrameric ESTs contained mt-sequences and since mtDNA was highly polymorphic, different mtDNA sequences (with access number provided if not the UCSC reference) were used as the reference to get the best match during sequence alignment. Therefore, the exact nt numbers and locations might slightly differ from different publications.

### RT-PCR, T-A cloning and DNA sequencing

We cultured HEK293 and Hela cells in the routine fashion and extracted total RNA from whole cell lysates with Trizol as described before [36], [37]. In some experiments, RNA samples were treated with Ambion® TURBO DNA-free™ Kit (Cat # AM1907, Life Technology), which has a much higher affinity to DNA than conventional DNase I, to remove DNA. RT of the total RNA to cDNA was performed using random hexamers, followed by PCR to amplify cDNA with primers specific to different ESTs. The novel, spliced mtRNA 16SrRNA-s was amplified with mtF2006 (5′-GGTGATAGCTGGTTGTCCAA-3′) as the forward primer and mtR3071 (5′-GAACTCAGATCACGTAGGAC-3′) as the reverse primer. All PCR products were fractioned in 0.9–1.2% agarose gel and visualized by ethidium bromide staining. The band of interest was excised out and purified using a simple method we described before [38]. The purified DNA fragment was cloned into a T-A vector and the resultant plasmids were sent to Genewiz (http://www.genewiz.com) for sequencing. The details of these methods were described before [15], [37].
